# Histological analysis of the intestinal wall of newborn rats submitted to hypoxia and reoxygenation to evaluate the protective effect of N-Acetylcysteine^1^

**DOI:** 10.1590/s0102-865020200040000001

**Published:** 2020-06-12

**Authors:** Soraia Guerra Silvares, Antonio Fernandes Moron, Manuel de Jesus Simões, Álvaro Ulhoa Cintra, Edna Frasson de Souza Montero, Edward Araujo, José Luiz Martins

**Affiliations:** I Fellow Master degree, Postgraduate Program in Obstetrics, Universidade Federal de São Paulo (UNIFESP), Brazil. Acquisition of data, manuscript writing, final approval.; II PhD, Department of Obstetrics, UNIFESP, Sao Paulo-SP, Brazil. Supervision, final approval.; III PhD, Department of Morphology and Genetics, UNIFESP, Sao Paulo-SP, Brazil. Analysis and interpretation of data, final approval.; IV PhD, Division of Pediatric Surgery, UNIFESP, Sao Paulo-SP, Brazil. Analysis and interpretation of data, final approval.; V PhD, Division of General and Trauma Surgery, Universidade de São Paulo (USP), Brazil. Critical revision final approval.; VI PhD, Department of Obstetrics, UNIFESP, Sao Paulo-SP, Brazil. Manuscript writing, final approval.; VII PhD, Division of Pediatric Surgery, UNIFESP, Sao Paulo-SP, Brazil. Conception and design of the study, final approval.

**Keywords:** Enterocolitis, Necrotizing, Hypoxia, Acetylcysteine, Rats

## Abstract

**Purpose:**

To evaluate the effect of N-Acetylcysteine (NAC) in newborn rats submitted to hypoxia and reoxygenation (H/R) conditions in an experimental model of necrotizing enterocolitis.

**Methods:**

Eight pregnant rats and their 70 cubs were used (5 groups) and exposed to H/R conditions and received NAC at different times. The animals in the H/R groups were placed in a gas chamber (100% CO^2^) for 10 minutes and then reoxygenated for 10 minutes (100% O^2^), twice a day for the first three days of life, with a six-hour span between events. On the third day of life, the animals were anesthetized, laparotomized and the intestines were resected.

**Results:**

The H/R and NAC groups showed changes in the intestinal wall in relation to the number, height and width of the villi when compared to the control group (p<0.0001), but with better preservation of structures in the NAC group. There were no differences between groups regarding the number (%) of mitoses.

**Conclusion:**

The administration of NAC decreased the lesions in the intestinal wall of rats submitted to H/R, therefore suggesting that this drug can be used to prevent the development of necrotizing enterocolitis in newborns.

## Introduction

Necrotizing enterocolitis (NEC) is the most common gastrointestinal disease in newborns, especially premature infants, with a significant rate of morbidity and mortality^1^. It is diagnosed between 2-7% of admissions to the neonatal Intensive Care Unit (ICU), with a mortality rate at between 10-50%, and the rapid progression form has a mortality of almost 100%^2^. Although NEC occurs more frequently in the first three months of life, 90% are low birth weight neonates with lower gestational ages at 28 weeks and 10% are newborns at term^1^.

NEC also represents an annual expenditure of approximately US $ 6.5 million in the United States; therefore, this is an important public health problem^3^. Advances in obstetric and neonatal care have improved the survival rate in low weight premature infants, consequently increasing the population at risk of developing NEC^4^.

Prematurity exposes the newborn to numerous causes of stress, such as hypotension, hypoxia, hypothermia, artificial feeding, anemia and umbilical catheterization^5^. These factors are causes of ischemic injuries of the neonatal intestine. It is believed that there is a circulatory deviation mechanism of blood perfusion of the noblest organs (brain, heart, and kidneys) at the expense of other admission organs, such as the intestine, leading to ischemic injury and necrosis^6^. It is also described that in addition to prematurity, hyperosmolar feeding, treatment with indomethacin, congenital heart disease, umbilical catheterization, polycythemia and pre-eclampsia are predisposing factors to NEC^7^.

Okur *et al*.^8^ and Özkan *et al*.^9^developed experimental models of NEC in which newborn rats submitted to hypoxia and reoxygenation associated with hypothermia presented intestinal lesions of NEC similar to those of human newborns. The models were reproduced and modified in the study by Meyer *et al*.^10^ and Cintra *et al*.^11^.

Inflammatory responses play an essential role in the pathogenesis of NEC. It is known that, in inflammatory processes, there is the formation of free oxygen radicals, which have been implied as mediators of intestinal ischemia-reperfusion (I/R)^12^. In this context, N-Acetylcysteine (NAC) becomes interesting due to its antioxidant action, which is reacting to free radicals through sulfhydryl groups (donating electrons) and transforming into cystine, or as a substrate for cystine synthesis (yielding cysteine). This property gave rise to several studies that seek to elucidate its effectiveness in prevention and treatment of injuries associated with the formation of free radicals in various organs, especially in phenomena involving oxidative stress^13-16^.

The limited therapy of NEC has stimulated researchers to seek adjuvant strategies, with the aim of preventing intestinal injuries. NAC is a low-cost, low-toxicity drug that has been used for over half a century in pulmonology and, since the mid-1970s, for the treatment of paracetamol poisoning^17^. There have also been several studies involving this drug, where it is evidenced NAC’s ability to protect organs such as the kidneys, heart and liver, due to its antioxidant capacity^18,19^.

The exogenous administration of free radical scavengers has been researched in order to reduce oxidative stress induced by H/R and antioxidants appear to be particularly useful^20^. Therefore, we consider studying the hypothesis that NAC would promote a decrease in hypoxia-induced intestinal lesions and neonatal reoxygenation. Based on the above, the purpose of the present study was to evaluate the protective effect of NAC on lesions on the intestinal wall of newborn rats submitted to H/R conditions, within the morphological parameters of the intestinal villi.

## Methods

This is an experimental, interventional, controlled and randomized study, assessed and approved by the Research Ethics Committee of the Universidade Federal de São Paulo (UNIFESP) (nº 0943/11). The study was conducted at the Pediatric Surgery Research Laboratory using pregnant outbred rats, of the Wistar EPM-1 lineage (*Rattus norvegicus*), provided by the Center for the Development of Experimental Models for Biology and Medicine at UNIFESP.

### *Management of animals during pregnancy*

Eight pregnant rats and their offspring were used, with four of these pregnant rats having received NAC 10% (Laboratório União Química, lot: 1327819), at a dose of 1.5 ml/kg, for five consecutive days before the expected date of delivery; the rest received nothing. The rats were kept under standard environmental conditions of day and night cycles, hygiene, temperature from 21 to 23°C, water ad libitum and specific ration for the species.

### *Management of animals after giving birth*

After 21 days, the rats gave birth to 70 pups, weighing between 5.5 and 7.5 grams, of both sexes, which were divided into five groups, and were kept in the maternal company throughout the experiment. After birth, the pups were submitted to hypoxia and reoxygenation (H/R), except for the control group.

### *Management of animals that received no drugs after giving birth*

The 39 newborn pups of both sexes from the 4 pregnant rats that received no drugs were randomly selected and separated into three groups: Group I (Control, n = 8, Average Weight AW= 7.2g), Group II (submitted to H/R and received no drugs before each event: H / R, n = 10, AW = 7.08 g) and Group III (submitted to H / R and NAC 10% intraperitoneally at the dose of 1,5ml/Kg: H/R - RN NAC, n = 21, AW = 7.04 g).

### *Management of animals that received NAC after giving birth*

**Figure 1:**
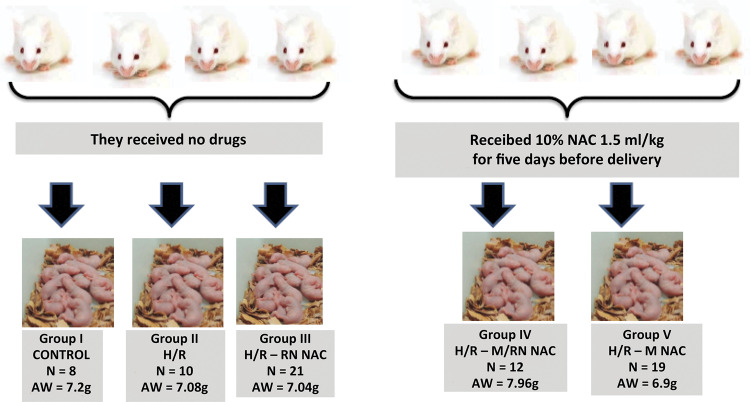
Diagram of the division of the groups studied.

The 31 newborn pups from the four pregnant rats that had received NAC were randomly selected and separated into two groups: Group IV (submitted to H / R and NAC 10% intraperitoneally at a dose of 1.5 ml / Kg: H / R- M / RN NAC, n = 12, AW = 7.96 g) and Group V (submitted to H / R and received no drugs: H / R-M NAC, n = 19, AW = 6.9 g). Figure 1 shows the scheme of the division of the groups studied. These rats received NAC because it is known that the pharmacokinetics are different in newborns and adults, and this fact can affect the result due to the different bioavailability of the drug.

### *General management of the pups*

The pups’ date of birth was determined by performing a vaginal smear of the rat on the day after copulation to identify the presence of sperm, counting an additional 20 days to the date of delivery.

In a special gas chamber (model CGS CO2 G - brand Beira-Mar - Brazil), the litters of groups II, III, IV and V were submitted to breathing for 10 minutes of 100% CO2 and then 10 minutes of O2 at 100%, with the flow of gases flow meter controlled to remain constant. This model depicts stress in the intestine, similar to that of children who develop NEC. A modified protocol presented in similar experiments was used^9-12^.

This hypoxia / reoxygenation (H/R) event was performed twice daily, at a six-hour span for three consecutive days (1st, 2nd and 3rd day of life) in all groups, except for the control group. 10% NAC was administered intraperitoneally to newborn rats (Groups III and IV) 15 minutes before being subjected to the conditions of the experiment. After the described procedures, the pups were kept in maternal company and at a temperature of 21 to 23ºC.

All pups were submitted to longitudinal laparotomy after intraperitoneal anesthesia with 0.1 ml of a mixture of anesthetics (0.2 ml of 10% ketamine plus 0.1 ml of 2% xylazine) 24 hours after the last H/R event. In laparotomy, the ileum portion was evaluated macroscopically after undergoing H/R, in order to evaluate edema, distension, perforation, necrosis or hemorrhage in the viscera. A portion of the ileum was removed for further histological analysis (mucosa, submucosa, vessels and muscle fibers) using the hematoxylin-eosin (HE) technique. The pathologist responsible for the histological analysis had no knowledge of which group each analyzed sample belonged to.

From each animal, a 1 cm fragment of the ileum was removed and submitted to standard histological technique (fixed in formal, buffered, dehydrated, embedded in paraffin and cut). The sections were mounted on slides and stained with hematoxylin-eosin for morphological examination of the mucosa, submucosa, vessels and muscle fibers. 06-micrometer sections were analyzed under optical microscopy by the same pathologist who was unaware of which group the fragment belonged to.

In the analysis of slides stained by the hematoxylin-eosin method, the optical binocular microscope was used, with a 400-fold magnification, and the intestinal villi were analyzed in relation to height, width and presence of mitoses.

For the analysis of the results, parametric or non-parametric tests were applied, taking into account the variables studied. Analysis of variance or Kruskal-Wallis analysis of variance was performed to compare the study groups; this was followed by the Tukey post hoc test that was performed to evaluate differences among groups, comparing Groups I and III and Groups V and VI. In all tests, the significance level set at 5% (p <0.05) was used.

## Results

**Figure 2:**
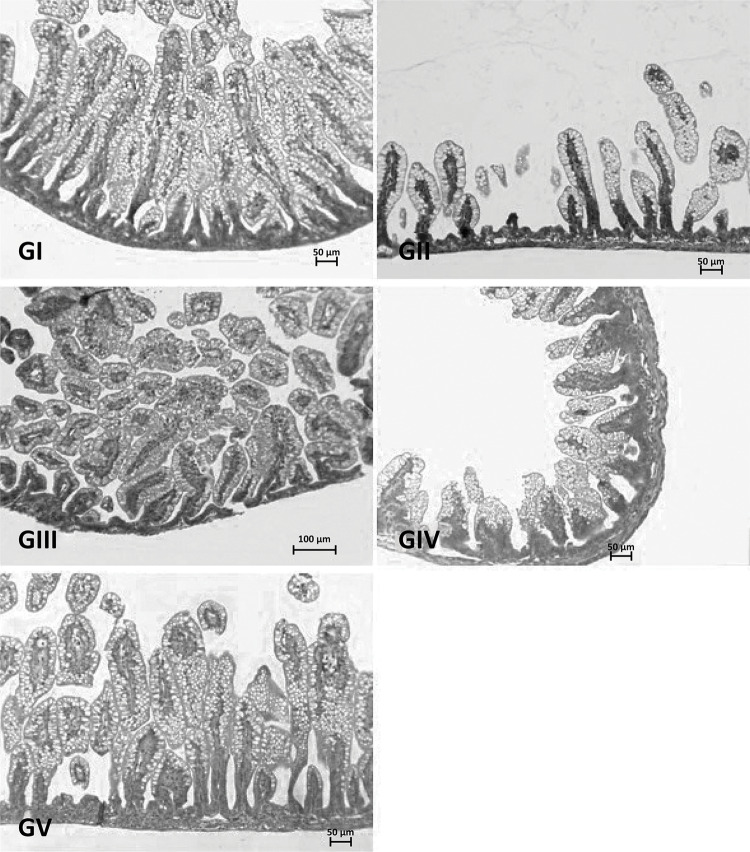
Photomicrographs of ileum sections of newborns belonging to the various study groups (GI, GII, GIII, GIV and GV). Note in the ischemic groups whose mothers and / or fetuses received higher villi NAC (GIII, GIV and GV) and in the group that did not receive N-acetylcysteine sparse villi (GII). H.E. 50µm bar.

In the control group (GI), we noticed in the small intestine numerous intestinal glands (Lieberkhun) and high villi lined by simple cylindrical epithelium where goblet and absorption cells (enterocytes) are noted. It was also observed, inside the villi, the presence of an axis of connective tissue rich in blood vessels (Fig. 2). In group II, we observed few villi as well as intestinal glands, the villi being covered by a simple cylindrical epithelium, consisting of goblet and absorption cells. It was also noted the presence of an axis of connective tissue rich in blood vessels, which were congested, in addition to numerous figures of mitoses in the luminal epithelium (Fig. 2). The results of Groups III and IV (H / R - RN NAC and H / R - RN / M NAC) showed a small intestine made up of countless intestinal glands and higher and thicker villi than in the ischemic group (GII). In these groups, the villi were lined with a simple cylindrical epithelium made up of goblet and absorption cells, in addition to the presence of a connective tissue axis rich in blood vessels (Fig. 2). In the ileum of newborn rats belonging to the group whose mothers and ischemic fetuses who had received NAC, a large concentration of villi was observed, being larger and wider (Fig. 2). In all groups, we noticed the presence of an axis of connective tissue rich in blood vessels. Table 1 shows the data regarding the number of villi in each group.

**Table 1:** Number of intestinal villi in each group.

	Group I Control	Group II H/R	Group III H/R – RN NAC	Group IV H/R- RN/ M NAC	Group V H/R- M NAC
	16.2	11.2	10.1	15.0	14.5
	15.1	10.3	12.3	12.1	12.7
	13.8	11.1	14.1	10.0	15.8
	14.1	12.1	10.3	11.1	14.2
	16.3	11.2	13.3	10.1	11.3
	15.7		11.1	12.2	16.5
	16.1		14.2	13.1	14.8
	15.5		13.1	12.2	16.7
			12.2	11.1	15.4
			10.1	13.1	16.7
			11.1	12.1	17.8
			13.1	10.2	18.9
			13.4		16.5
			10.1		15.4
			12.2		16.3
			12.0		
			12.1		
			11.1		
			10.1		
**Median Average**	**15.6 15.4**	**11.2 11.2**	**12.1 11.9**	**12.1 11.9**	**15.8 15.6**

Kruskal-Wallis analysis of variance: Calculated H = 34.84 (p<0.0001)

Post hoc test: Group I > Group II and Group III. Group IV = Group V

In Group V (H / R - M NAC), that received NAC during pregnancy before the hypoxia / reoxygenation (H/R) procedure, the variable referring to the number of villi, after the statistical tests that were performed, presented p-value with differences when compared with Group II (H/R) and Group III (H/R - RN NAC) and there were no differences when compared to the control group. Table 2 shows the data regarding the villus height of each group.

**Table 2:** Height (µm) of the intestinal villi of newborns in each group.

	Group I Control	Group II H/R	Group III H/R – RN NAC	Group IV H/R- M e RN NAC	Group V H/R- M NAC
	333.55	190.1	233.12	224.40	315.70
	309.12	180.3	324.50	232.00	316.20
	320.39	195.4	312.60	111.00	317.80
	322.41	220.1	223.11	202.30	318.70
	321.11	190.1	243.21	201.00	314.20
	319.12		222.31	221.70	300.50
	311.18		221.34	202.40	320.40
	328.20		245.30	203.40	317.50
			223.40	201.3	318.40
			222.10	202.3	300.50
			321.12	203.4	301.20
			300.12	199.1	320.40
			199.76		330.50
			189.12		340.20
			190.87		320.1
			301.43		
			321.23		
			212.21		
			221.12		
**Median Average**	**320.75 320.71**	**190.1 195.2**	**223.40 248.84**	**202.90 208.69**	**317.80 316.82**

Kruskal-Wallis analysis of variance: Calculated H = 37.55 (p<0.0001)

Post hoc test: Group I > Group III > Group II. Group V > Group IV.

We also observed that in Group V (H/R - M NAC) the variable referring to the height of the villi showed a p-value with difference when compared to Group IV (H/R - RN / M NAC) and Group II (H/R). The villus height, in the control group, showed a p-value with a difference when compared to the other groups studied. It is possible to conclude that, in relation to the number and height of the villi, the administration of NAC during pregnancy has a more expressive protective factor when compared to the administration in newborn rats or when no drugs are administered. Table 3 shows the data regarding the villus width of each group.

**Table 3:** Width (µm) of the intestinal villi of each group.

	Group I Control	Group II H/R	Group III H/R –RN NAC	Group IV H/R- RN/ M NAC	Group V H/R- M NAC
	12.55	6.2	*68.34*	48.10	12.50
	19.60	5.3	52.31	50.20	11.40
	21.50	4.2	60.34	49.00	10.50
	22.30	4.5	62.34	47.10	11.20
	28.60	6.2	54.12	47.20	12.40
	23.20		87.12	48.10	13.50
	19.10		57.14	51.00	12.40
	16.20		62.34	52.00	11.50
			61.12	53.10	10.50
			60.35	55.10	12.40
			54.34	52.00	11.50
			84.56	44.50	12.60
			67.89		11.70
			45.67		10.70
			56.71		11.70
			57.12		
			67.12		
			67.12		
			66.34		
**Median Average**	**20.30 19.79**	**5.30 5.21**	**61.12 62.04**	**49.60 49.62**	**11.70 11.74**

Kruskal-Wallis analysis of variance: Calculated H = 52.85 (p<0.0001)

Post hoc test: Group III > Group I > Group II. Group IV > Group V

Regarding the variable width of the intestinal villi, Group III (H/R - RN NAC) showed a p value with difference when compared to Group II (H/R) and Group V (H/R - M NAC). Group IV (H/R - RN / M NAC) presented p-value with differences when compared to Group II (H/R) and Group V (H/R - M NAC). Table 4 shows the data regarding the number of mitoses in the villi of each group.

**Table 4:** Number (%) of mitoses in the intestinal villi of each group.

	Group I Control	Group II H/R	Group III H/R –RN NAC	Group IV H/R- RN/ M NAC	Group V H/R- M NAC
	2.1	3.7	*3.1*	1.0	1.3
	3.1	1.4	3.2	1.0	1.4
	1	1.9	2.1	1.1	1.5
	2.9	2.6	1.1	2.0	1.2
	2.1	2.7	1.0	2.1	1.1
	3.2		1.1	1.0	1.4
	1.1		2.1	2.5	1.5
	2.2		2.1	3.3	1.6
			2.2	2.5	1.7
			1.2	1.2	1.8
			1.1	1.0	1.9
			4.2	2.1	1.9
			1.1		1.3
			1.2		1.4
			1.3		1.5
			1.5		
			1.3		
			1.1		
			1.1		
**Median Average**	**2.15 2.21**	**2.60 2.46**	**1.30 1.74**	**1.60 1.73**	**1.50 1.50**

Kruskal-Wallis analysis of variance

Calculated H = 4.66 (P = 0.1979)

Not significant

When comparing the number of mitoses present in each group, there were no differences between them, indicating that although we were able to observe differences between the groups’ intestinal villus morphometries, there were no degrees of injuries that would generate an increase in tissue mitosis. Table 5 shows the mortality rate in the groups studied.

**Table 5:** Mortality rate in the study groups.

	Group I Control	Group II H/R	Group III H/R –RN NAC	Group IV H/R- RN/ M NAC	Group V H/R- M NAC
Total	8	10	*21*	12	19
Deaths	0	5	2	0	4
Survivors	8	5	19	12	15
%	0	50	9.52	0	21.5

## Discussion

We now know that antioxidants play an important role in pathophysiological processes, due to the large number of reactive oxygen species generated during the processes of aging, cancer, inflammation, among other situations. Researchers have demonstrated positive results in the use of NAC, specifically in the situation of ischemia and reperfusion, namely: hemorrhagic shock^21^, hepatic ischemia^16^_,_ muscular compartment syndrome^22^, acute renal failure^18^, among others. This same protective role was found when analyzing ischemia and reperfusion in the intestine^19,20,23^.

Likewise, the administration of NAC in pregnant animals showed protective effects on fetuses^24^. The anti-inflammatory activity of NAC in the amniotic fluid, which crosses the placental barrier, promotes an increase in fetal hepatic glutathione. Thus, there is great potential for the use of NAC in the treatment of pregnant women with infectious and inflammatory processes, which can induce the fetal growth restriction and premature birth and be a risk factor for NEC. The results obtained in the experiment also demonstrated the protective action of NAC in the groups that received the treatment. We used the height and width of the villi as a reference to verify this protection, in addition to the number of mitoses figures found. However, in the latter, we found no significant difference between the groups, most likely related to the low degree of tissue damage obtained.

In some experimental studies of ischemia and reperfusion^11,12,15^, the histopathological references used to measure protection were the classification of intestinal injury according to Chiu *et al*.^25^ and Ozdemir *et al*.^23^, who used their own classification as parameters for histological evaluation. Montero *et al*.^15^ used the score proposed by Park *et al*.^26^ In the present study, we defined using the width and height of the intestinal villi obtained as parameters for the histological evaluation of the different groups.

Other investigative researches used biochemical markers (products of the lipid peroxidation that identifies cell destruction, such as tissue malonaldehyde, D-lactate, nitric oxide and others) as references to assess the degree of injury^10,11,15,23,27^.

We observed, in the different experiments, that the degree of injury found depends on the conditions of the experiment, such as time of hypoxia and reoxygenation, exposure of animals to hypothermia, feeding with mother or artificial milk and maintenance of the calf with the parent. In the present study, the maintenance of the young rats with their mothers and feeding with breast milk showed a degree of protection for the litter.

The first description of the NEC model in newborn rats was reported by Barlow *et al*.^28^, who induced the disease with the combination of artificial feeding, followed by intermittent episodes of stress due to hypoxia and a day of hypothermia, which went from three to four days and standardized the use of ischemia using nitrogen. Dvorak *et al*.^29^ used the same model, but for four days and Meyer *et al*.^10^, as well as Cintra *et al*.^11^, reproduced the model by Okur *et al*.^8^ who for three consecutive days submitted the newborn rats to periods of hypoxia and reoxygenation, but without artificial feeding and hypothermia. The highest degrees of injury obtained take place when there is more time for hypoxia and reoxygenation, more time for exposure to hypothermia and the presence of artificial feeding.

In the present study, the same conditions of the study by Ozdemir *et al*.^23^ were used, but we kept the litters with the parents receiving breast milk. This factor was considered preponderant for not obtaining such significant injuries, given that the artificial diet is considered one of the risk factors in the pathogenesis of NEC. Due to this fact, we used the morphological characteristics of the intestinal villi as parameters for comparison.

The animals were sacrificed on the third day or 72 hours after the beginning of the experiment, since Caplan *et al*.^30^ demonstrated a high mortality rate in newborn rats after the fourth day.

This study allows us to conclude that we can use NAC in a preventive manner, before delivery, to protect the newborn from NEC, in those patients who are at greater risk of preterm birth. The literature shows that NAC decreases the production of cytokines in the placenta, and may also inhibit premature delivery^24^. The result also suggests greater protection when NAC is administered during pregnancy. This fact probably occurs because it is a prodrug, and NAC must be metabolized to become active. In newborns, the metabolization process is still under development, limiting the production of active metabolites. This explains the lesser protection when NAC is administered only to newborns, using the same reference dose of the mothers.

In addition, this study demonstrated that NAC has a protective effect against the development of NEC in the fetus during pregnancy. At the same time, this preventive administration can contribute to inhibit premature birth, which is one of the factors related to the development of this disease, in addition to other complications that may arise for the newborn and the genitor.

The number of animals in each group was limited and different because the number of animals that were born in the previous two groups was different (rats that received drugs and rats that did not receive drugs), therefore, there is the possibility of type 1 error.

## Conclusions

N-Acetylcysteine, when administered, protected the newborn rats’ intestinal walls submitted to H/R. In addition, newborn rats whose parents had received NAC prior to delivery had greater protection than those who received NAC only after birth but due to the great variety of elements that can impact the results obtained, it is interesting that there are more studies on the topic.

## Footnotes

1 Research performed at Department of Obstetrics, Universidade Federal de São Paulo (UNIFESP), Brazil. Part of Master degree thesis, Postgraduate Program in Obstetrics, UNIFESP. Tutor: Prof. José Luiz Martins.

## References

[1] Schnabl KL, Van Aerde JE, Thomson AB, Clandinin MT (2008). Necrotizing enterocolitis: a multifactorial disease with no cure. World J Gastroenterol.

[2] Neu J, Weiss MD (1999). Necrotizing Enterocolitis: pathophysiology and prevention. JPEN J Parenter Enteral Nutr.

[3] Patole S (2005). Prevention of necrotizing enterocolitis: year 2004 and beyond. J Matern Fetal Neonatal Med.

[4] Lin PW, Stoll BJ (2006). Necrotizing Enterocolitis. Lancet.

[5] Alajbegovic-Halimic J, Zvizdic D, Alimanovic-Halilovic E, Dodik I, Duvnjak S (2015). Risk factors for retinopathy of prematurity in premature born children. Med Arch.

[6] Barbiro-Michaely E, Tolmasov M, Rinkevich-Shop S, Sonn J, Mayevsky A (2007). Can the “brain-sparing effect” be detected in a small-animal model?. Med Sci Monit.

[7] Samuels N, van de Graaf RA, Jonge RCJ, Reiss IKM, Vermeulen MJ (2017). Risk factors for necrotizing enterocolitis in neonates: a systematic review of prognostic studies. BMC Pediatr.

[8] Okur H, Kucukaydin M, Kose K, Kontas O, Dogam P, Kazez A (1995). Hypoxia-induced necrotizing enterocolitis in the immature rat: the role of lipid peroxidation and management by vitamin E. J Pediatr Surg.

[9] Özkan KU, Özokutan BH, Ínanç F, Boran C, Kilinç M (2005). Does maternal nicotine exposure during gestation increase the injury severity of small intestine in the newborn rats subjected to experimental necrotizing enterocolitis. J Pediatr Surg.

[10] Meyer KF, Martins JL, Freitas LG, Oliva ML, Patrício FR, Macedo M, Wang L (2006). Evaluation of an experimental model of necrotizing enterocolitis in rats. Acta Cir Bras.

[11] Cintra AEU, Martins JL, Patricio FRS, Higa SEM, Montero EFS (2008). Nitric oxide in the intestines of mice submitted to ischemia and reperfusion: L-arginine effects. Transplant Proc.

[12] Lloyd JR (1969). The etiology of gastrointestinal perforation in the newborn. J Pediatr Surg.

[13] Didoné EC, Cerski CT, Kalil N (2002). N-acetylcysteine decreases liver congestion in ischemia and reperfusion injury - experimental study. Rev Col Bras Cir.

[14] Rodrigues AJ, Évora PR, Schaff HV (2004). Protective effect of N-acetylcysteine against oxygen radical-mediated coronary artery injury. Braz J Med Biol Res.

[15] Montero EF, Abrahão MS, Koike MK, Manna MC, Ramalho CE (2003). Intestinal ischemia and reperfusion injury in growing rats: hypothermia and n-acetylcysteine modulation. Microsurgery.

[16] Salim CS, Montero EF, Simões MJ, Abrahão MS, Ramalho CE, Fagundes DJ (2002). Effect of N-acetylcysteine on the lung after liver ischemia in rats. Acta Cir Bras.

[17] Rodrigues AJ, Évora PB, Schaff HV (2004). Protective effect of N-acetylcysteine against oxygen radical-mediated coronary artery injury. Braz J Med Biol Res.

[18] Nitescu N, Ricksten SE, Marcussen N, Haraldsson B, Nilsson U, Basu S, Guron G (2006). N-acetylcysteine attenuates kidney injury in rats subjected to renal ischaemia-reperfusion. Nephrol Dial Transplant.

[19] Azeredo MA, Azeredo LA, Eleuthério EC, Schanaider A (2008). Propofol and N- Acetylcysteine attenuate oxidative stress induced by intestinal ischemia/reperfusion in rats. Protein carbonyl detection by immunoblotting. Acta Cir Bras.

[20] Hazinedaroglu SM, Dulger F, Kayaoglu HA, Pehlivan M, Serinsoz E, Canbolat O, Erverdi N (2004). N-Acetylcysteine in intestinal reperfusion injury: an experimental study in rats. ANZ J Surg.

[21] Portella AO, Montero EF, Poli de Figueiredo LF, Bueno AS, Thurow AA, Rodrigues FG (2004). Effects of N-acetylcysteine in hepatic ischemia-reperfusion injury during hemorrhagic shock. Transplant Proc.

[22] Kearns SR, O’Briain DE, Sheehan KM, Kelly C, Bouchier-Hayes D (2010). N-acetylcysteine protects striated muscle in a model of compartment syndrome. Clin Orthop Relat Res.

[23] Ozdemir R, Yurttutan S, Sarı FN, Uysal B, Unverdi HG, Canpolat FE, Erdeve O, Dilmen U (2012). Antioxidant effects of N-acetylcysteine in a neonatal rat model of necrotizing enterocolitis. J Pediatr Surg.

[24] Awad N, Khatib N, Ginsberg Y, Weiner Z, Maravi N, Thaler I, Ross MG, Itsokovitz-Eldor J, Beloosesky R (2011). N-acetyl-cysteine (NAC) attenuates LPS-induced maternal and amniotic fluid oxidative stress and inflammatory responses in the preterm gestation. Am J Obstet Gynecol.

[25] Chiu CJ, McArdle AH, Brown R, Scott HJ, Gurd FN (1970). Intestinal mucosa lesion in low-flow states. Arch Surg.

[26] Park PO, Haglund U, Bulkley GB, Fält K (1990). The sequence of development of intestinal tissue injury after strangulation ischemia and reperfusion. Surgery.

[27] Cuzzocrea S, Mazzon E, Costantino G, Serraino I, De Sarro A, Caputi AP (2000). Effects of n-acetylcysteine in a rat model of ischemia and reperfusion injury. Cardiovasc Res.

[28] Barlow B, Santulli TV (1975). Importance of multiple episodes of hypoxia or cold stress on the development of enterocolitis in animal model. Surgery.

[29] Dvorak B, Halpern M, Holubec H, Williams CS, McWilliam DL, Dominguez JA, Stepankova R, Payne CM, McCuskey RS (2003). Epidermal growth factor reduces the development of necrotizing enterocolitis in a neonatal rat model. Am J Physiol Gastrointest Liver Physiol.

[30] Caplan MS, Hedlund E, Adler L, Hsueh W (1994). Role of asphyxia and feeding in a neonatal rat model of necrotizing enterocolitis. Pediatric Pathol.

